# Intraoperative estimation of liver boundary conditions from multiple partial surfaces

**DOI:** 10.1007/s11548-023-02964-5

**Published:** 2023-06-01

**Authors:** Andrea Mendizabal, Eleonora Tagliabue, Diego Dall’Alba

**Affiliations:** Department of Computer Science, University of Verona, Verona, Italy

**Keywords:** Patient-specific simulation, Intraoperative model update, Augmented surgery, Boundary conditions

## Abstract

**Purpose:**

A computer-assisted surgical system must provide up-to-date and accurate information of the patient’s anatomy during the procedure to improve clinical outcome. It is therefore essential to consider the tissue deformations, and a patient-specific biomechanical model (PBM) is usually adopted. The predictive capability of the PBM is highly influenced by proper definition of attachments to the surrounding anatomy, which are difficult to estimate preoperatively.

**Methods:**

We propose to predict the location of attachments using a deep neural network fed with multiple partial views of the intraoperative deformed organ surface directly encoded as point clouds. Compared to previous works, providing a sequence of deformed views as input allows the network to consider the temporal evolution of deformations and to handle the intrinsic ambiguity of estimating attachments from a single view.

**Results:**

The method is applied to computer-assisted hepatic surgery and tested on both a synthetic and in vivo human open-surgery scenario. The network is trained on a patient-specific synthetic dataset in less than 5 h and produces a more accurate intraoperative estimation of attachments than applying the ones generally used in liver surgery (i.e., fixing vena cava or falciform ligament). The obtained results show 26% more accurate predictions than other solution previously proposed.

**Conclusions:**

Trained with patient-specific simulated data, the proposed network estimates the attachments in a fast and accurate manner also considering the temporal evolution of the deformations, improving patient-specific intraoperative guidance in computer-assisted surgical systems.

**Supplementary Information:**

The online version contains supplementary material available at 10.1007/s11548-023-02964-5.

## Introduction

During a complex computer-assisted surgery, the integration and effective visualization of preoperative data allow the surgeon to access the information available on the specific patient, which can improve the operative outcome [1, 2]. In the context of hepatic surgery, the surgeon needs to continuously keep track of several structures of interest (e.g., tumors to be removed or blood vessels to be preserved), which move with respect to their preoperative location due to liver deformations induced by surgical manipulations and interactions with the surrounding tissues.

The possibility to rely on deforming liver would provide valuable intraoperative assistance to the surgeon, who can continuously monitor the up-to-date location of the critical structures. In order to meet the accuracy requirements of hepatic surgery, such augmented view must be built from a patient-specific biomechanical model (PBM) which accounts for patient-specific characteristics (e.g., anatomy, mechanical properties and boundary conditions) and image data (e.g., liver’s deformed visible surface) [3]. This PBM model can support not only advanced visualization techniques, but also registration methods between pre-operative and intra-operative data, able to handle the presence of anatomical deformations even when landmark or fiducial correspondences are not available.

The finite-element (FE) method is generally used to solve such PBM relying on continuum mechanics laws, for its accuracy and ability to simulate a wide range of materials. The FE formulation requires the definition of material properties (e.g., Poisson’s ratio and Young’s modulus for elastic materials) and boundary conditions [4]. While the Poisson’s ratio is generally set to 0.5 to model tissues incompressibility, other material parameters like the Young’s modulus vary with age and pathology, and may require patient-specific estimation, for instance based on elastographic techniques [5] or by exploiting Kalman filtering [2]. However, estimation of material properties is not necessary in a scenario as the one considered in this work, where surface constraints are imposed, so the effect of Young’s modulus can be neglected [6].

When the input is represented by surface constraints, the so-called Dirichlet boundary conditions (BCs) play a major role in the deformation of the considered object [4, 6, 7] and need to be carefully estimated. They correspond to the attachment points of the organ to the surrounding anatomy, and they are usually not visible or hard to estimate from the preoperative images. Hence, in order to provide the surgeon with an up-to-date augmented view reflecting the intraoperative conditions of the patient, such attachment points should be characterized intraoperatively [2, 4, 7]. A few works have addressed the intraoperative estimation of BCs in the context of hepatic surgery. Authors in [1, 4] propose to estimate the location of the falciform ligament (i.e., one of the main anatomical liver attachment) by initializing their model with a statistical atlas, which is sensitive to inter-patient variations. As an alternative, authors in [8] propose to use additional intraoperative sensors, which can be complex to introduce into clinical practice. Moreover, data acquired intraoperatively introduces uncertainty to the system which can be addressed with Bayesian filtering as proposed in [7, 9], where authors use Kalman filters to estimate the attachments of the liver as stochastic parameters. However, such filters are very sensitive to the initialization parameters which can make the algorithm fail in specific conditions.

A recent approach [10] has introduced a deep neural network called BA-Net (binary-attachment network) that estimates adipose tissue’s BCs intraoperatively, starting from a single intraoperative point cloud of the deformed adipose tissue, without relying on any *a priori* assumption about their location. Authors have proposed a complete pipeline which has shown capable of very fast intraoperative estimation of organ attachment points [11], guaranteeing the PBM to be continuously up-to-date, thus reflecting changes due to surgical manipulation. Inspired by this work, we propose an extended version of BA-Net, called BA-Net 2.0, applied for BCs estimation in a different context than adipose tissue manipulation (i.e., liver surgery), which introduces the following three main contributions:

- First of all, original BA-Net is trained on various random anatomical shapes, which makes it usable for any patient geometry but at the cost of a loss in accuracy and very long training times. Instead, we choose here to rely on a patient-specific network trained only with synthetic data built from the patient’s geometry. This allows for short training times (only a few hours), compatible with the standard surgical workflow.
- Secondly, one partial surface deformation is in general not sufficient to fully characterize the BCs of a model, in particular when the attachment area is hidden. Indeed, several BCs can satisfy the given input surface deformation without being mechanically meaningful (non-uniqueness of the solution to the inverse problem). In order to reduce the amount of possible BCs, we propose here to feed BA-Net with multiple non-consecutive views describing significantly different deformations allowing the network to better understand the attachments and to account for time evolution.
- Thirdly, the original BA-Net is fed with the displacement of the visible points, which needs an extra step for estimating the correspondence between the points of the intraoperative point cloud and those of the model. This step may introduce approximation errors and can be very sensitive to the quality of the initial rigid registration of the model and the surgical data. In this work, we overcome this limit by feeding directly the network with the raw intraoperative point cloud, letting it implicitly learn points-to-surface correspondences, thus reducing possible sources of error.

## Methods

BA-Net 2.0 is a 3D convolutional neural network that predicts a binary mask of attachment points, given multiple pieces of deformed surfaces as input. As previously described, the difficulty of knowing the attachment points results in the lack of real annotated datasets. This lack is not only due to the extensive manual annotation work required, but also because not even expert surgeons know with precision how and where the liver is attached to the surrounding organs for each specific patient. Hence, we train the network with synthetic samples produced with a patient-specific FE model. The synthetic dataset used, composed of samples with random deformation and attachments, allows to simulate all possible intraoperative conditions, thus allowing the network to learn the association between deformations and BCs. In Sect. 2.1, the patient-specific training data generation process is described in detail. In Sect. 2.2, we present the BA-Net 2.0 approach to incorporate multiple frames, describing the differences with previous BA-Net. In Sect. 2.3, the overall pipeline for the intraoperative update of BCs is depicted.

### Patient-specific training data generation

We generate a synthetic data set using the FE method, similarly to [10] and [11]. Instead of generating the synthetic dataset based on random meshes, we propose to rely on a patient-specific geometry obtained from the preoperative CT scan to generate the training dataset. Furthermore, the organ surface geometry is embedded in a regular grid, and only the cells inside the organ or those overlapping its boundary are kept in order to generate a hexahedral mesh for the FE simulation [3]. This choice is not only motivated for the stability and good accuracy of hexahedral meshes, but also because it matches the required grid structure as input for BA-Net 2.0. Moreover, it allows us to directly use the surface models obtained from the preoperative CT scans without the need of tetrahedral meshing. We model the liver as a Neo-Hookean material [12] with Poisson’s ratio of 0.48 and Young’s modulus of 5000 *Pa*.

The variability of the dataset relies on the imposed constraints, on the attachments and on the visible surface. For each sample, random attachment points are generated by extracting an irregular subset of surface points that can cover between 5 and 50 % of the surface. The attachment region includes points around a randomly sampled node depending on a metric that combines distance to the sampled point and noise, as described in details in [10]. Once the attachment points are defined, we simulate the tissue manipulation by applying a force of random magnitude and direction over a small random area of the surface of the organ. Newton–Raphson algorithm is used to solve for the displacements of the model given such randomly chosen constraints. We repeat the simulated tissue manipulation *F* times to generate *F* different deformations associated to the same set of attachments (*F* stands for the number of non-consecutive frames). Then, for each deformation, a partial piece of liver surface is extracted to mimic the partial field of view of the surgical context, following an approach similar to the generation of the attachment points. The extracted visible surface ranges between 10 and 100% of the entire liver surface, considering a fixed virtual camera point of view.

Once the synthetic dataset is generated, the physical quantities of interest are encoded in an $n_x \times n_y \times n_z$ grid structure as follows. The deformed visible surfaces are represented using *F* distance fields $(DF_i)_{i=1...F}$, and are given as input to the network [13]. The attachments are encoded as a binary mask *M*, where 1 means “fully constrained point” and 0 means “unconstrained point.” Such mask *M* represents the output of the network. Thus, our training dataset is composed of *N* samples of pairs $\left( [DF_1, DF_2,..., DF_F], M\right)$ where each $(DF_i)_{i=1...F}$ is an array of size $1\times n_x \times n_y \times n_z$ and *M* is a binary mask of size $1\times n_x \times n_y \times n_z$. The same process is followed to generate a testing data set.

To ensure the quality of the dataset, only numerically stable samples are kept, i.e., simulations for which the Newton–Raphson algorithm converges to a solution. Moreover, samples exceeding 0.3 *m* of L2 displacement are removed.

### BA-Net 2.0

**Fig. 1:**
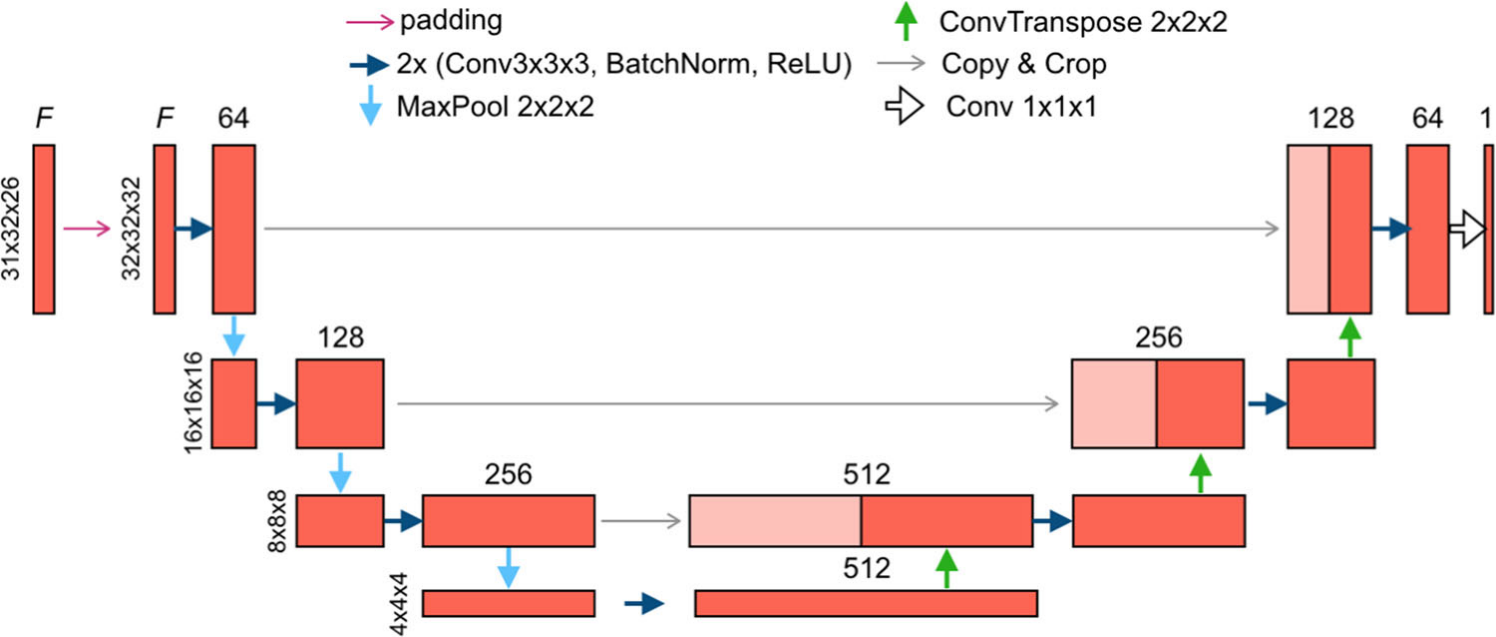
BA-Net 2.0 architecture. F stands for the number of input frames

BA-Net 2.0 is a 3D U-shaped convolutional neural network, the result of a sequence of max-pooling and up-sampling operations (Fig. 1). Since it applies standard convolutions, it requires a grid-like structure as input. Differently from previous works [10, 11], where a regular $64^3$ grid was used, BA-Net 2.0 relies on a $31 \times 32 \times 26$ grid ($n_x=31$, $n_y=32$, $n_z=26$), where each dimension fits at best the 3D size of the considered object. This was possible because BA-Net 2.0 is a patient-specific network, and the liver dimensions are known beforehand. Note that the grid cell size matches the FE mesh cell size (used for data generation), allowing for a one-to-one correspondence between the two topologies.

The input of the original BA-Net is composed by the displacement of the nodes of a piece of visible surface interpolated to the 3D grid using a Gaussian kernel (three-dimensional array), together with the object geometry encoded as a signed distance field in the grid, thus leading to an input of size $4 \times 64^3$. On the contrary, BA-Net 2.0 is patient-specific so it does not need the surface geometry in the input. Instead, it is directly fed with the sequence of *F* frames representing *F* deformed surface point clouds. Each frame contains the nodal positions of the partial deformed visible surface encoded as a distance field in the $31 \times 32 \times 26$ grid, thus leading to an input of size $F \times 31 \times 32 \times 26$.

The output of the network is the binary mask of attachment points *M*. This choice is consistent with the representation proposed in the original BA-Net; the only difference relies on the one-to-one correspondence between the fixed points in the mesh and the ones in the grid. This makes it immediate to apply the BCs predicted by the network in the PBM update.

BA-Net 2.0 architecture is well-established in the biomechanical simulation field [2, 14, 15] obtaining speed and accuracy suitable for surgical application, thus demonstrating its capacity to learn the necessary information.

As training loss, we chose a combination of the dice similarity coefficient (DSC) and the binary cross-entropy (BCE) as done in [10] with a batch size equal to 5. The network is trained using the AdamW optimizer [16] on a workstation with AMD Ryzen 7 3700X CPU and NVIDIA 2080Ti GPU.

**Fig. 2:**
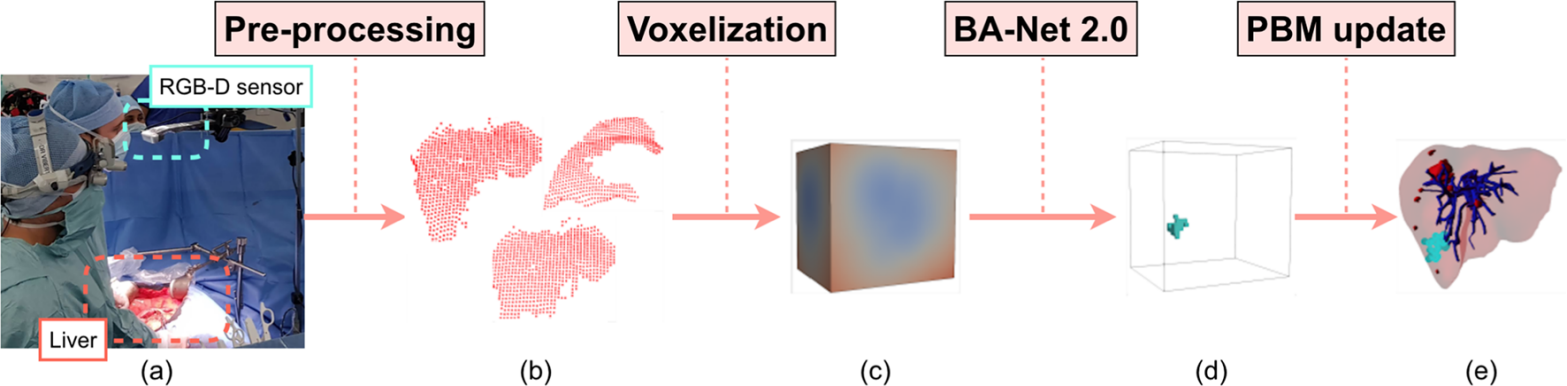
Intra-operative update of attachment points during open liver surgery using BA-Net 2.0. **a** Experimental setup. Point clouds of liver surface are acquired during surgery with an RGB-D sensor. **b** Surface point clouds of the deformed liver. **c** Voxelized point clouds encoded as distance fields in the 3D grid of resolution $n_x \times n_y \times n_z$. **d** Attachment points estimated by BA-Net 2.0 (cyan) in grid format. (e) Intra-operative PBM with updated attachments, as predicted by BA-Net 2.0

### Intraoperative update of BCs

The pipeline of our approach is shown in Fig. 2 for an hepatic open surgery scenario. The tri-dimensional surface information is acquired with an RGB-D sensor placed anywhere above the patient, as shown in Fig. 2a. The acquired point clouds are processed with both spatial and color segmentation to extract the current view of the deformed liver from the full anatomical view (visible in Fig. 2b). In particular, we used the Grabcut segmentation algorithm as done in [17], in order to visually segment the liver from the RGB image. Then, the obtained segmentation is used as a binary mask to extract the point cloud of the region of interest from the depth map recorded by the depth sensor. A manual registration is performed once at the beginning of the surgery to align the acquired data with the PBM, based on geometric features visible in both models and known spatial relationships (e.g., the fixed position of the RGB-D sensor with respect to the patient). A sequence of surface point clouds of the deformed liver are then encoded as distance fields (Fig. 2c) and used directly as input to BA-Net 2.0. In the original BA-Net approach, the input included the displacement of the visible points of the object. Obtaining such displacement from a depth map describing the deformed surface of the organ requires an extra step for finding the correspondences between points. Regardless of the method used to compute correspondences, this is the main bottleneck of the previous BA-Net, both in terms of accuracy and computation time, as demonstrated in [11]. This step is removed in the proposed pipeline, thus simplifying it and skipping the most time consuming part. Instead, we rely on BA-Net 2.0 for implicitly learning correspondences between points, which makes the method more suitable for on-line intraoperative use. The prediction obtained from BA-Net 2.0 visible in Fig. 2d is then applied to update the PBM (Fig. 2e). This step is straightforward thanks to adopted input and output encoding that guarantee a one-to-one correspondence.

## Experiments and results

In order to validate BA-Net 2.0, we propose two main sets of experiments on a liver geometry. On the one hand, we evaluate its performance on a synthetic dataset and explore the main differences between the original BA-Net [10] and the current one. On the other hand, we will apply BA-Net 2.0 to human data acquired during an open liver surgery at Paul Brousse Hospital in Paris. For both scenarios, we will consider one single liver geometry obtained from the patient’s preoperative CT scan, collected at Paul Brousse Hospital. When groundtruth attachments are available (i.e., for the synthetic scenario), we will compute the dice similarity coefficient (DSC) to the network’s prediction. For the human data set, we will instead compute target registration errors on visible surfaces. The method and collected datasets are available at https://gitlab.com/altairLab/banet/-/tree/banet2.0.

### Synthetic liver manipulation

We generated a training data set of 5263 valid samples following the strategy described in “Patient-specific training data generation” Section with $F=1$, $F=2$ and $F=3$ non-consecutive frames, in less than 12 h (around $8\,s$ per sample). The network was trained in less than 5 h which makes the approach compatible with clinical constraints, as the data can be generated and the network trained over-night. Each prediction takes $23.07 \pm 2.21 ~ ms$, which allows for a real-time update of the BCs.

We compared the accuracy of the network for different number of non-consecutive frames and different types of input (visible surface positions or displacements), by computing DSC. Obtained results are shown in Table 1. It is not surprising that the accuracy of BA-Net 2.0 (i.e., using only visible surface positions as input) when using a single-input deformed state ($F=1$) is lower than the one obtained with the original BA-Net (i.e., using the displacement of the visible surface points as input), when using the same amount of training parameters and data. Indeed, learning the BCs from the positions of the visible surface is more complex than learning them from the displacement of the visible points, as the network needs to learn the correspondences between the points as well. However, reliance on positions instead of displacements facilitates on-line intraoperative use (see “BA-Net 2.0” Section).

When considering multiple frames ($F=3$), there is an improvement of 26% when compared to $F=1$. Note that if the multiple frames are generated from the same grasping point, there is no significant improvement when compared to one single frame. Hence, for each sample, we generated various deformations with different force amplitudes and application points. Moreover, if the input deformations are significantly different, increasing the number of frames above 3 gives a relatively small improvement compared to the extra time needed to generate these extra frames.

**Table 1:** Comparison of the average dice score (DSC) obtained over a testing data set of 100 synthetic samples, for different number of non-consecutive frames as input (*F*), and different types of input displacements for BA-Net, while positions for BA-Net 2.0

Method	F	DSC (mean ± std)
BA-Net	1	$0.54 \pm 0.21$
BA-Net 2.0	1	$0.39 \pm 0.25$
BA-Net 2.0	3	$0.49 \pm 0.23$

**Fig. 3:**
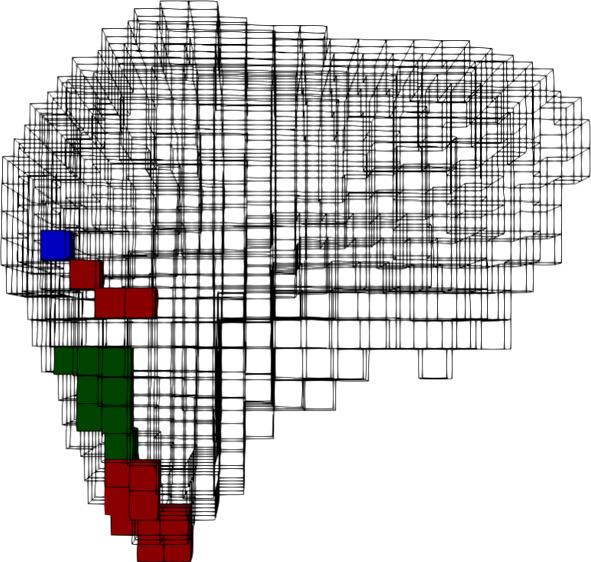
BA-Net 2.0 predicted attachments with one frame as input ($F=1$). Prediction appears in red for frame $F_1$, in blue for frame $F_2$ and in green for frame $F_3$. The overlap (given as a DSC with respect to prediction made with $F_1$) is equal to $0.42 \pm 0.41$

**Fig. 4:**
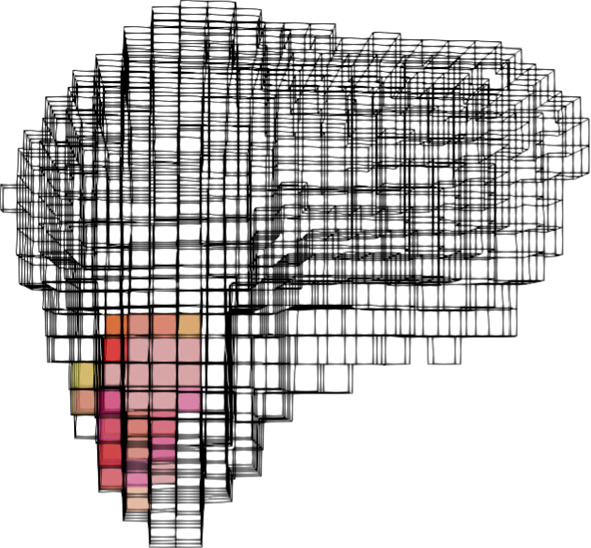
BA-Net 2.0 predicted attachments with three frames as input ($F=3$) ordered in the six possible configurations ($(F_1, F_2, F_3)$, $(F_1, F_3, F_2)$, $(F_2, F_1, F_3)$, $(F_2, F_3, F_1)$, $(F_3, F_1, F_2)$ and $(F_3, F_2, F_1)$. The overlap (given as a DSC with respect to the first prediction) is equal to $0.88 \pm 0.06$

### In vivo liver manipulation

In this section, we will apply BA-Net 2.0 to real data from a human liver surgery as shown in Fig. 2. The liver is manipulated from different grasping points, inducing various levels of deformation. The collected point clouds are filtered such that samples with large occlusions and with deformations exceeding that of training range are removed (at maximum $0.3\,m$ of L2 displacement is allowed).

At the beginning of the surgery, we collected three frames with considerably different deformations and gave them to BA-Net 2.0 trained with the synthetic dataset (“Patient-specific training data generation” Section). Figures 3 and 4 show the predictions when using 1 and 3 frames, respectively. Predictions obtained relying on one input frame only ($F=1$) are completely different from one another (there is almost no overlap between the three predicted attachments), which reflects the difficulty of the problem to be solved and the non-uniqueness of the solution. On the other hand, the prediction made with three frames is insensitive to the order of the frames as shown by the big overlap between the predictions. In the following, we consider the attachments predicted by BA-Net 2.0 with frames $(F_1, F_2, F_3)$ given in temporal order.

Since the groundtruth attachments are not exactly known, we validate our approach by comparing the result of the intraoperative elastic registration task aiming at deforming the preoperative liver model to align it with the current surgical view. The elastic registration is constrained by different set of attachments.

Due to the lack of volumetric information during this surgery, the outcome of the registration is assessed by comparing the real point cloud with the outcome of the registration. In particular, the acquired point cloud is split into two subsets. The subset of points corresponding to the most deformed area (around $15\%$ of the point cloud) is used to drive the FEM registration (i.e., it represents the target position of the corresponding points in the FEM). The remaining points are used to assess the accuracy of the registration, thus they are considered as ground truth positions to compute nodal L2 errors with the predicted deformed states (output of the registration). In our evaluation, we rely on the same FE model described for the training data generation (“Patient-specific training data generation” Section). However, it is not required that the model is the same (e.g., the mesh might have higher resolution).

**Fig. 5:**
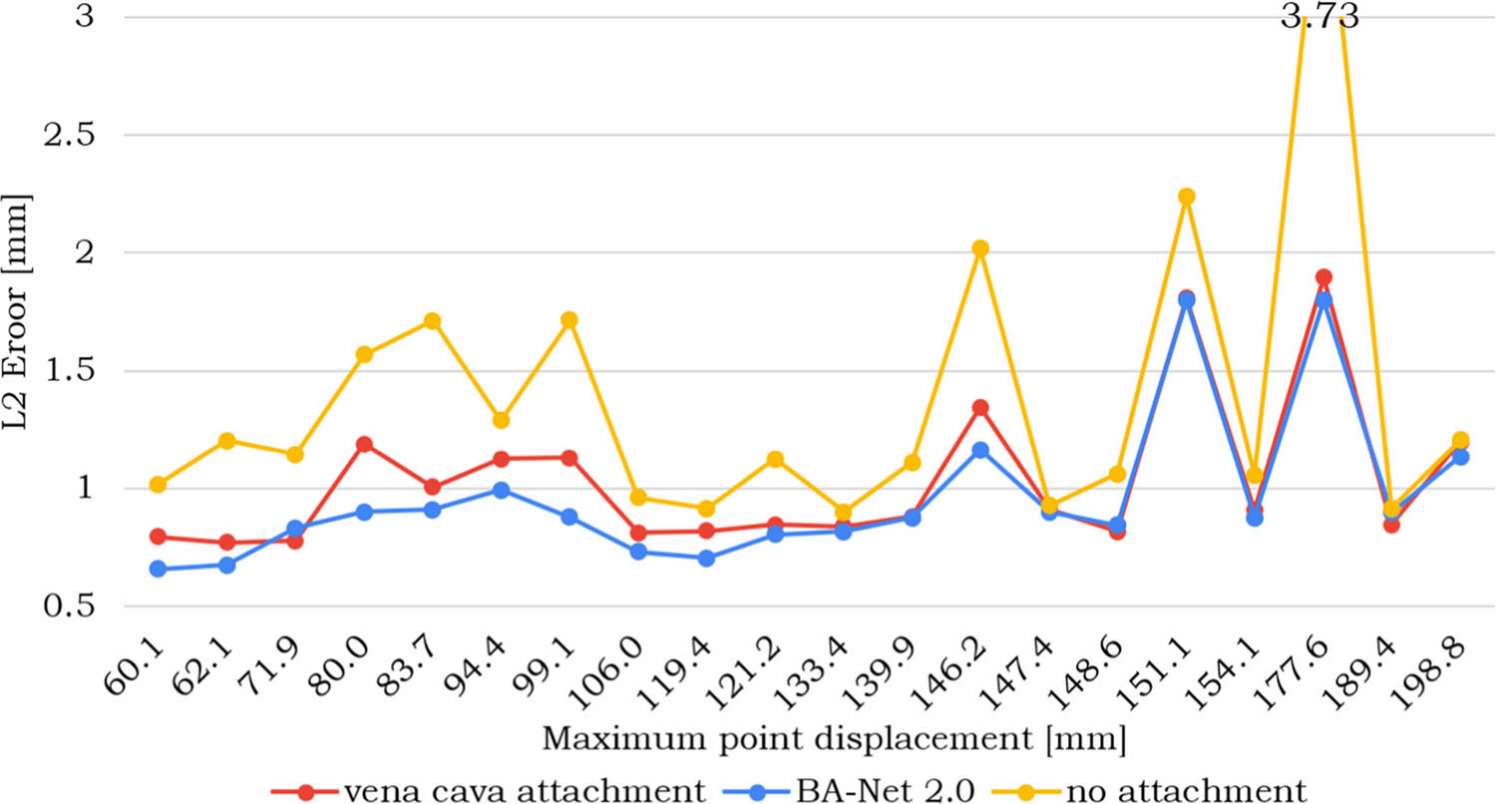
Comparison of *in vivo* FEM registration using *vena cava* fixations, BA-Net 2.0 predicted attachments with multiple frames, or no attachments at all. Nodal L2 errors are given in *mm* for 20 samples and are ordered in increasing deformation amounts

When providing deformable models for hepatic surgery, it is a common practice to constrain the liver around *vena cava* which is the stiffer part of the organ [4]. For this reason, we considered the registration results obtained with *vena cava* attachments as a baseline reference. In Table 2, the distributions of the obtained nodal L2 errors are given for the reference *vena cava* fixations and for the BA-Net 2.0 predicted attachments with 1 or 3 frames as input. For completeness, we also added the results obtained with no attachments which correspond to the worse performance. The lowest error is provided by the model with attachments predicted with multiple frames ($F=3$) and is under 1 *mm* as median value, which satisfies clinical requirements [2]. The results obtained by the method with multiple frames ($F=3$) are also statistically significantly different ($p-value<0.05$) with respect to the other conditions considered in Table 2. Statistical analysis was conducted using an two-sided Wilcoxon rank sum test, since the samples are not normally distributed according to Lilliefors test. More exhaustively, the obtained nodal L2 errors for all the samples are displayed in the graph of Fig. 5. The error with BA-Net 2.0 predicted attachments is smaller than that produced with baseline fixations for all the samples but three. For these three samples, the difference in performance with respect to *vena cava* fixation is very small.

**Table 2:** Distribution of *in vivo* nodal target registration errors in *mm* for different type of attachments. Statistically significant differences (according to a two-sided Wilcoxon rank sum test) are indicated by a single asterisk if $p < 0.05$ or by two asterisks if $p < 0.01$

Type of attachments	L2 error (Median–IQR)
$\emptyset$**	1.19–0.60
vena cava*	0.95–0.33
BA-Net 2.0 $F_1$*	0.99–0.17
BA-Net 2.0 $F_2$*	0.99–0.38
BA-Net 2.0 $F_3$*	0.98–0.18
**BA-Net 2.0** $\varvec{{\textit{F}}=3}$	$\varvec{0.87} \varvec{\pm } \varvec{0.12}$

The bold font indicates the best results obtained

## Discussion and conclusion

All in all, we proposed a method for the patient-specific update of boundary conditions that can be trained in only a few hours and that produces a more accurate PBM than using baseline attachments. To do this, we encoded the intraoperative surfaces as distance fields (the displacement at each point does not need to be computed as in previous BA-Net, which reduces approximation errors), and we accounted for some temporal evolution by including non-consecutive frames in the input (which reduced the amount of possible solutions). Moreover, considering that BA-Net was originally proposed for estimating the BCs of adipose tissues and now it is applied for hepatic surgery, we can conclude that relying on deep neural networks is a promising approach for intraoperative parameters estimation in general.

One of the main limitations of the presented work relies on the data generation process that needs to take place for every new patient. The main bottleneck is explained by the randomness of the constraints (applied forces and attachments) that lead to a high number of non-valid samples (non-convergence of the Newton–Raphson solver). Future work will consider using modal analysis as proposed in [18] in order to produce meaningful sets of forces, leading to significantly different deformations. This would considerably reduce data generation times. Moreover, even if the modelization results appear to be improved with the proposed approach, the shape of the predicted attachments is quite surprising. Indeed, they are not anatomically meaningful as we know that the hepatic organ has free lobes with very few constraints. Yet, the augmented model is improved when fixing this area, which showcases the difficulty of the problem that we are trying to solve. Hence, as an improvement of the method, we will force BA-Net 2.0 to predict anatomically meaningful attachments by adapting our training data sets. Indeed, we stayed very generic in the data generation process, without exploiting all the *a priori* knowledge available on hepatic surgery. Instead of generating random fixations, we will start from a statistical atlas of the liver attachments to which we will add some randomness to still allow for unforeseen events. Also, instead of generating random visible surfaces, we will adapt them to the surgical scenario where we know that only between 10 and 50 % of the surface of the organ will be visible.

## Supplementary information

Additional data are given in Online Resource 1.

## Supplementary Information

Below is the link to the electronic supplementary material.

Supplementary file 1 (pdf 215 KB)
