# Supplementary material for: Intraoperative estimation of liver boundary conditions from multiple partial surfaces
Source: Int J Comput Assist Radiol Surg. 2023 Jun 1;18(7):1295–302. doi: 10.1007/s11548-023-02964-5 (PMC10329628; doi:10.1007/s11548-023-02964-5)
Supplement: Supplementary file 1 — (pdf 215 KB) [file 11548_2023_2964_MOESM1_ESM.pdf]

Intraoperative Estimation of Liver Boundary Conditions from Multiple Partial Surfaces

Andrea Mendizabal[1*], Eleonora Tagliabue[1] and Diego Dall'Alba[1]

[1]Dept. of Computer Science, University of Verona, Verona, Italy.

*Corresponding author(s). E-mail(s): an.men.ec@gmail.com;

Supplementary material

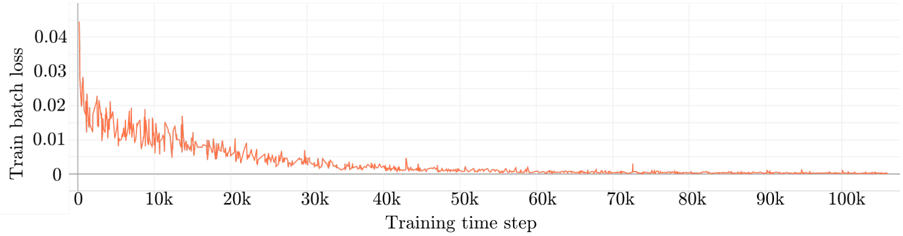

Fig. 1 Training loss curve. The loss used to train the network is a linear combination between the Dice similarity coefficient and binary cross entropy $L = \frac{1}{N}\sum_1^N(1 - DSC + BCE)$.
